# Supplementary material for: Is paternal age associated with transfer day, developmental stage, morphology, and initial hCG-rise of the competent blastocyst leading to live birth? A multicenter cohort study
Source: PLoS One. 2022 Jul 28;17(7):e0270664. doi: 10.1371/journal.pone.0270664 (PMC9333207; doi:10.1371/journal.pone.0270664)
Supplement: S1 Fig — 1Danish medical data center, 2Preimplantation genetic testing, 3Personal identification number, 4Ultrasound testing for pregnancy, 5Danish medical birth register. (DOCX) [file pone.0270664.s001.docx]

**S1 Fig. Flow chart**

7246

match in the database

DMDC^1^ Formatex (2014-2018)

Exclusion of, n=1326

(PGT^2^, oocytdonation and semen

donation)

5920

Exclusion of invalid CPR^3^, n=146

(Wrong number - and order of

digits and CPR^3^ from abroad)

Exclusion of positive 1. scan^4^

and negative 2. scan, n=328

5446

Loss to follow up, n=334

(Births outside study period,

births abroad, stillbirths and

abortions)

5112

match between a valid

CPR^3^ and a birth in

DMBR^5^ (2014-March 2019)

Exclusion of, n=241

(Births of twins and second birth)

Missing date for oocyte pick up

so age could not be calculated, n=29

4842

men and competent blastocyst

^1^Danish medical data center, ^2^Preimplantation genetic testing, ^3^Personal identification number, ^4^Ultrasound testing for pregnancy,

^5^Danish medical birth register
