# Supplementary figures and images for: Is paternal age associated with transfer day, developmental stage, morphology, and initial hCG-rise of the competent blastocyst leading to live birth? A multicenter cohort study
Source: PLoS One. 2022 Jul 28;17(7):e0270664. doi: 10.1371/journal.pone.0270664 (PMC9333207; doi:10.1371/journal.pone.0270664)

**S2 Fig. The correlation between women’s age and men’s age at oocyte pick up**


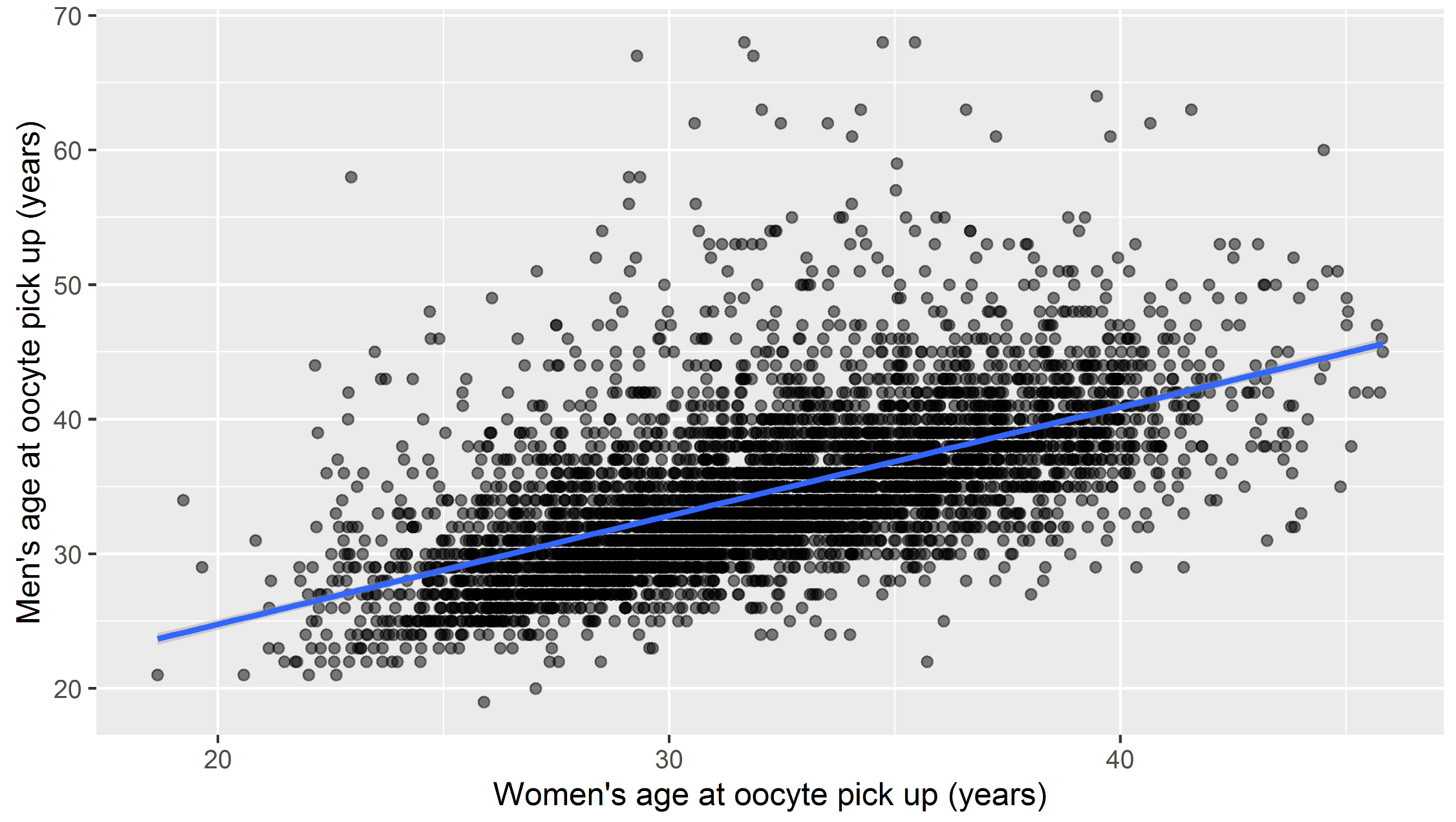

Supplement: S2 Fig — (DOCX) [file pone.0270664.s002.docx]
