# Supplementary material for: Is paternal age associated with transfer day, developmental stage, morphology, and initial hCG-rise of the competent blastocyst leading to live birth? A multicenter cohort study
Source: PLoS One. 2022 Jul 28;17(7):e0270664. doi: 10.1371/journal.pone.0270664 (PMC9333207; doi:10.1371/journal.pone.0270664)
Supplement: S1 Table — *Paternal age at oocyte pick up, 1Unexplained: Couples with unexplained infertility, 2Other: Female infertility caused by conditions in ovary, uterus, cervix or caused by other conditions (hepatitis, habitual abortion, asymptomatic HIV), 3IVF: In Vitro Fertilization, 4ICSI: Intracytoplasmic Sperm Injection, 5Day 5 blastocyst: For COS defined as blastocyst transfer at day 5 and for FET defined as cryopreservation at day 5, 6Day 6 blastocyst: For COS defined as blastocyst transfer at day 6 and For FET defined as cryopreservation at day 6, 7First measurement of serum human chorionic gonadotrophin (hCG). (DOCX) [file pone.0270664.s003.docx]

**S1 Table. Male age, treatment, blastocyst timing, morphology and implantation characteristics**

| **Age *(year)** | **20-24** | **25-29** | **30-34** | **35-39** | **40-45** | **46-70** |
| --- | --- | --- | --- | --- | --- | --- |
| **Age*, n (%)** | 80 (1.7) | 853 (17.6) | 1733 (35.8) | 1315 (27.2) | 635 (13.1) | 226 (4.7) |
| **BMI (kg/m^2^), mean (sd)** | 24.8 (3.5) | 25.5 (3.6) | 26.0 (3.9) | 26.2 (3.8) | 26.4 (3.7) | 26.9 (4.1) |
| **Smoking, cigarettes/day, n (%)** |  |  |  |  |  |  |
| 0 | 44 (77.2) | 541 (82.1) | 1117 (86.1) | 819 (82.9) | 404 (82.6) | 143 (82.7) |
| 1-5 | 1 (1.7) | 29 (4.4) | 62 (4.8) | 58 (5.9) | 38 (7.8) | 4 (2.3) |
| 6-10 | 7 (12.3) | 37 (5.6) | 57 (4.4) | 43 (4.3) | 24 (4.9) | 13 (7.5) |
| 11-20 | 5 (8.8) | 49 (7.4) | 52 (4.0) | 62 (6.3) | 22 (4.5) | 12 (6.9) |
| >20 | 0 (0) | 3 (0.5) | 9 (0.7) | 6 (0.6) | 1 (0.2) | 1 (0.6) |
| missing | 23 | 194 | 436 | 327 | 146 | 53 |
| **Female partners parity, n (%)** |  |  |  |  |  |  |
| 1 | 62 (84.9) | 668 (85.6) | 1169 (74.3) | 731 (62.5) | 364 (63.7) | 146 (74.1) |
| 2 | 10 (13.7) | 100 (12.9) | 358 (22.7) | 365 (31.2) | 170 (29.8) | 42 (21.3) |
| ≥3 | 1 (1.4) | 12 (1.5) | 47 (3.0) | 74 (6.3) | 37 (6.5) | 9 (4.6) |
| missing | 7 | 73 | 159 | 145 | 64 | 29 |
| **Indication for cause of infertility, n (%)** |  |  |  |  |  |  |
| anovulation | 9 (11.4) | 147 (17.5) | 226 (13.2) | 118 (9.1) | 43 (6.9) | 15 (6.8) |
| tube factor | 7 (8.9) | 63 (7.5) | 155 (9.1) | 118 (9.1) | 47 (7.5) | 15 (6.8) |
| endometriosis | 1 (1.3) | 43 (5.1) | 92 (5.4) | 60 (4.7) | 20 (3.2) | 8 (3.6) |
| male factor | 43 (54.4) | 322 (38.3) | 579 (33.9) | 436 (33.9) | 228 (36.5) | 92 (41.4) |
| unexplained^1^ | 17 (21.5) | 233 (27.8) | 568 (33.2) | 434 (33.6) | 191 (30.5) | 64 (28.8) |
| other^2^ | 2 (2.5) | 32 (3.8) | 89 (5.2) | 124 (9.6) | 96 (15.4) | 28 (12.6) |
| missing | 1 | 13 | 24 | 25 | 10 | 4 |
| **Number of previous transfers, n (%)** |  |  |  |  |  |  |
| 0 | 35 (43.8) | 348 (40.8) | 711 (41.0) | 488 (37.1) | 214 (33.7) | 84 (37.2) |
| 1 | 29 (36.3) | 264 (30.9) | 453 (26.1) | 346 (26.3) | 175 (27.6) | 63 (27.9) |
| 2 | 13 (16.3) | 118 (13.8) | 255 (14.7) | 194 (14.8) | 88 (13.9) | 25 (11.1) |
| 3 | 2 (2.5) | 69 (8.1) | 140 (8.1) | 114 (8.7) | 50 (7.9) | 23 (10.2) |
| ≥4 | 1 (1.1) | 54 (6.4) | 174 (10.1) | 173 (13.1) | 108 (16.9) | 31 (13.6) |
| **Fertilization method, n (%)** |  |  |  |  |  |  |
| IVF^3^ | 34 (42.5) | 402 (47.1) | 828 (47.8) | 615 (46.8) | 252 (39.7) | 75 (33.2) |
| ICSI^4^ | 46 (57.5) | 451 (52.9) | 905 (52.2) | 700 (53.2) | 383 (60.3) | 151 (66.8) |
| **Timing, n (%)** |  |  |  |  |  |  |
| day 5  blastocyst^5^ | 72 (90.0) | 761 (89.2) | 1507 (87.0) | 1117 (84.9) | 531 (83.6) | 194 (85.8) |
| day 6  blastocyst^6^ | 8 (10.0) | 92 (10.8) | 226 (13.0) | 198 (15.1) | 104 (16.4) | 32 (14.2) |
| **Stage, n (%)** |  |  |  |  |  |  |
| 1 | 1 (1.2) | 9 (1.1) | 22 (1.3) | 14 (1.1) | 6 (0.9) | 1 (0.4) |
| 2 | 0 (0) | 7 (0.7) | 14 (0.8) | 15 (1.1) | 6 (0.9) | 2 (0.9) |
| 3 | 9 (11.3) | 139 (16.4) | 269 (15.5) | 209 (15.9) | 90 (14.2) | 38 (16.8) |
| 4 | 47 (58.7) | 469 (54.9) | 929 (53.6) | 706 (53.8) | 350 (55.2) | 120 (53.1) |
| 5 | 21 (26.3) | 200 (23.4) | 441 (25.5) | 326 (24.8) | 152 (23.9) | 59 (26.1) |
| 6 | 2 (2.5) | 30 (3.5) | 58 (3.3) | 44 (3.3) | 31 (4.9) | 6 (2.7) |
| **Trophectoderm (TE), n (%)** |  |  |  |  |  |  |
| A | 46 (59.7) | 458 (56.4) | 988 (60.1) | 780 (62.6) | 380 (63.5) | 138 (63.6) |
| B | 30 (40.0) | 327 (40.3) | 607 (36.9) | 424 (34.0) | 202 (33.7) | 72 (33.2) |
| C | 1 (1.3) | 27 (3.3) | 50 (3.0) | 42 (3.4) | 17 (2.8) | 7 (3.2) |
| missing | 3 | 41 | 88 | 69 | 36 | 9 |
| **Inner cell mass (ICM), n (%)** |  |  |  |  |  |  |
| A | 49 (63.6) | 497 (61.2 | 1010 (61.4) | 817 (65.6) | 392 (65.4) | 139 (64.1) |
| B | 26 (33.8) | 294 (36.2) | 593 (36.0) | 396 (31.8) | 189 (31.6) | 76 (35.0) |
| C | 2 (2.6) | 21 (2.6) | 42 (2.6) | 33 (2.6) | 18 (3.0) | 2 (0.9) |
| missing | 3 | 41 | 88 | 69 | 36 | 9 |
| **hCG^7^, mean (sd)** | 392.0 (214.9) | 417.6 (252.5) | 406.7 (258.9) | 394.2 (252.4) | 410.7 (255.7) | 450.0 (298.9) |

^*^Paternal age at oocyte pick up, ^1^Unexplained: couples with unexplained infertility, ^2^Other: female infertility caused by conditions in ovary, uterus, cervix or caused by other conditions (hepatitis, habitual abortion, asymptomatic HIV), ^3^IVF: In Vitro Fertilization, ^4^ICSI: Intracytoplasmic Sperm Injection, ^5^Day 5 blastocyst: For COS defined as blastocyst transfer at day 5 and for FET defined as cryopreservation at day 5, ^6^Day 6 blastocyst: For COS defined as blastocyst transfer at day 6 and For FET defined as cryopreservation at day 6, ^7^First measurement of serum human chorionic gonadotrophin (hCG).
