# Supplementary material for: Is paternal age associated with transfer day, developmental stage, morphology, and initial hCG-rise of the competent blastocyst leading to live birth? A multicenter cohort study
Source: PLoS One. 2022 Jul 28;17(7):e0270664. doi: 10.1371/journal.pone.0270664 (PMC9333207; doi:10.1371/journal.pone.0270664)
Supplement: S2 Table — One-way ANOVA. *Paternal age at oocyte pick up, 1COS: Controlled Ovarian Stimulation, 2TE: Trophectoderm, 3ICM: Inner Cell Mass, 4Group 1: 6AA, 6BA, 5AA, 5BA, 4AA, 4BA, Group 2: 6AB, 6BB, 6CB, 6CA, 5AB, 5BB, 5CB, 5CA, 4AB, 4BB, 4CB, 4CA, Group 3: 6AC, 6BC, 6CC, 5AC, 5BC, 5CC, 4AC, 4BC, 4CC, 3AA, 3AB, 3BA, 3AC, 3CA, 3BB, 3BC, 3CB, 3CC, 2AA, 2AB, 2BA, 2AC, 2CA, 2BB, 2BC, 2CB, 2CC, 1AA, 1AB, 1BA, 1AC, 1CA, 1BB, 1BC, 1CB, 1CC. (DOCX) [file pone.0270664.s004.docx]

**S2 Table. The unadjusted association of paternal age^*^ with day of transfer, developmental stage and morphology of the competent blastocyst after COS^1^**

|  | **N** | **Mean (sd)** | **Meandiff. (95%CI)** | **P overall** | **P trend across categories** |
| --- | --- | --- | --- | --- | --- |
| **Age^*^** | 2044 | 34.68 (5.96) |  |  |  |
| **Transfer day** | 2044 |  |  | **-** | **-** |
| **5** | 2001 | 34.63 (5.94) | ref. |  |  |
| **6** | 43 | 36.81 (6.39) | 2.18 (0.38;3.98) |  |  |
| **Stage** | 1979 |  |  | 0.08 | 0.94 |
| **3** | 289 | 35.14 (6.01) | ref. |  |  |
| **4** | 1212 | 34.51 (5.89) | -0.63 (-1.40;0.13) |  |  |
| **5** | 461 | 34.73 (6.15) | -0.41 (-1.29;0.47) |  |  |
| **6** | 17 | 37.53 (5.05) | 2.39 (-0.53;5.31) |  |  |
| missing | 65 |  |  |  |  |
| **TE^2^** | 1959 |  |  | 0.96 | 0.91 |
| **A** | 1301 | 34.69 (5.92) | ref. |  |  |
| **B** | 593 | 34.70 (6.11) | 0.01 (-0.56;0.60) |  |  |
| **C** | 65 | 34.48 (5.42) | -0.21 (-1.70;1.28) |  |  |
| missing | 85 |  |  |  |  |
| **ICM^3^** | 1959 |  |  | 0.85 | 0.76 |
| **A** | 1343 | 34.70 (5.97) | ref. |  |  |
| **B** | 561 | 34.70 (6.0) | -0.001 (-0.59;0.59) |  |  |
| **C** | 55 | 34.24 (5.56) | -0.46 (-2.07;1.15) |  |  |
| missing | 85 |  |  |  |  |
| **Group^4^** | 1959 |  |  | 0.49 | 0.51 |
| **1** | 1199 | 34.66 (5.94) | ref. |  |  |
| **2** | 441 | 34.50 (6.06) | -0.16 (-0.81;0.49) |  |  |
| **3** | 319 | 35.02 (5.91) | 0.36 (-0.38;1.09) |  |  |
| missing | 85 |  |  |  |  |

t – test. One-way ANOVA. ^*^Paternal age at oocyte pick up, ^1^COS: Controlled Ovarian Stimulation, ^2^TE: Trophectoderm, ^3^ICM: Inner Cell Mass, ^4^Group 1: 6AA, 6BA, 5AA, 5BA, 4AA, 4BA, Group 2: 6AB, 6BB, 6CB, 6CA, 5AB, 5BB, 5CB, 5CA, 4AB, 4BB, 4CB, 4CA, Group 3: 6AC, 6BC, 6CC, 5AC, 5BC, 5CC, 4AC, 4BC, 4CC, 3AA, 3AB, 3BA, 3AC, 3CA, 3BB, 3BC, 3CB, 3CC, 2AA, 2AB, 2BA, 2AC, 2CA, 2BB, 2BC, 2CB, 2CC, 1AA, 1AB, 1BA, 1AC, 1CA, 1BB, 1BC, 1CB, 1CC.
