# Supplementary material for: Is paternal age associated with transfer day, developmental stage, morphology, and initial hCG-rise of the competent blastocyst leading to live birth? A multicenter cohort study
Source: PLoS One. 2022 Jul 28;17(7):e0270664. doi: 10.1371/journal.pone.0270664 (PMC9333207; doi:10.1371/journal.pone.0270664)
Supplement: S3 Table — One-way ANOVA. *Paternal age at oocyte pick up, 1FET: Frozen-thawed Embryo Transfer, 2TE: Trophectoderm, 3ICM: Inner Cell Mass, 4Group 1: 6AA, 6BA, 5AA, 5BA, 4AA, 4BA, Group 2: 6AB, 6BB, 6CB, 6CA, 5AB, 5BB, 5CB, 5CA, 4AB, 4BB, 4CB, 4CA, Group 3: 6AC, 6BC, 6CC, 5AC, 5BC, 5CC, 4AC, 4BC, 4CC, 3AA, 3AB, 3BA, 3AC, 3CA, 3BB, 3BC, 3CB, 3CC, 2AA, 2AB, 2BA, 2AC, 2CA, 2BB, 2BC, 2CB, 2CC, 1AA, 1AB, 1BA, 1AC, 1CA, 1BB, 1BC, 1CB, 1CC. (DOCX) [file pone.0270664.s005.docx]

**S3 Table. The unadjusted association of paternal age^*^ with day of transfer, developmental stage and morphology of the competent blastocyst after FET^1^**

|  | **N** | **Mean (sd)** | **Meandiff. (95%CI)** | **P overall** | **P trend across categories** |
| --- | --- | --- | --- | --- | --- |
| **Age^*^** | 2798 | 34.48 (6.11) |  |  |  |
| **Cryopreservation day** |  |  |  | **-** | **^-^** |
| **5** | 2181 | 34.34 (6.23) | ref. |  |  |
| **6** | 617 | 34.98 (5.65) | 0.64 (0.09;1.19) |  |  |
| **Stage** | 2766 |  |  | 0.91 | 0.83 |
| **3** | 465 | 34.34 (5.98) | ref. |  |  |
| **4** | 1409 | 34.53 (6.33) | 0.19 (-0.45;0.83) |  |  |
| **5** | 738 | 34.54 (5.91) | 0.20 (-0.51;0.91) |  |  |
| **6** | 154 | 34.31 (5.65) | -0.03 (-1.14;1.09) |  |  |
| missing | 32 |  |  |  |  |
| **TE^2^** | 2637 |  |  | **0.01** | **0.01** |
| **A** | 1489 | 34.78 (6.18) | ref. |  |  |
| **B** | 1069 | 34.03 (6.07) | -0.75 (-1.23;-0.26) |  |  |
| **C** | 79 | 34.65 (6.22) | -0.13 (-1.52;1.26) |  |  |
| missing | 161 |  |  |  |  |
| **ICM^3^** | 2637 |  |  | 0.10 | **0.04** |
| **A** | 1561 | 34.68 (6.14) | ref. |  |  |
| **B** | 1013 | 34.17 (6.17) | -0.51 (-1.0;-0.03) |  |  |
| **C** | 63 | 34.10 (5.51) | -0.58 (-2.14;0.96) |  |  |
| missing | 161 |  |  |  |  |
| **Group^4^** | 2637 |  |  | **0.02** | 0.07 |
| **1** | 1369 | 34.77 (6.13) | ref. |  |  |
| **2** | 771 | 34.0 (6.25) | -0.77 (-1.31;-0.23) |  |  |
| **3** | 497 | 34.40 (5.98) | -0.37 (-1.0;.0.26) |  |  |
| missing | 161 |  |  |  |  |

t – test. One-way ANOVA. ^*^Paternal age at oocyte pick up, ^1^FET: Frozen-thawed Embryo Transfer, ^2^TE: Trophectoderm, ^3^ICM: Inner Cell Mass, ^4^Group 1: 6AA, 6BA, 5AA, 5BA, 4AA, 4BA, Group 2: 6AB, 6BB, 6CB, 6CA, 5AB, 5BB, 5CB, 5CA, 4AB, 4BB, 4CB, 4CA, Group 3: 6AC, 6BC, 6CC, 5AC, 5BC, 5CC, 4AC, 4BC, 4CC, 3AA, 3AB, 3BA, 3AC, 3CA, 3BB, 3BC, 3CB, 3CC, 2AA, 2AB, 2BA, 2AC, 2CA, 2BB, 2BC, 2CB, 2CC, 1AA, 1AB, 1BA, 1AC, 1CA, 1BB, 1BC, 1CB, 1CC.
