# Supplementary material for: Is paternal age associated with transfer day, developmental stage, morphology, and initial hCG-rise of the competent blastocyst leading to live birth? A multicenter cohort study
Source: PLoS One. 2022 Jul 28;17(7):e0270664. doi: 10.1371/journal.pone.0270664 (PMC9333207; doi:10.1371/journal.pone.0270664)
Supplement: S6 Table — Linear regression. Multiple linear regression. *Men’s age at oocyte pick up, **Adjusted for female age, female BMI, female smoking, diagnosis and clinic, 1human chorionic gonadotrophin, 2FET: Frozen-thawed Embryo Transfer. (DOCX) [file pone.0270664.s008.docx]

**S6 Table. The association of men’s age^*^ with implantation, initial hCG^1^ rise, of the competent blastocyst after FET^2^ – without 526 first transfers**

| **Women age^*^ (years)** | **N** | **Missing** | **Mean hCG (sd)** | **Meandiff. (95%CI)** | **P-value** | **Adj. meandiff.**  **(95%CI)^**^** | **P-adj** |
| --- | --- | --- | --- | --- | --- | --- | --- |
| **21-24** | 34 | 4 | 424.3  (248.6) | -33.9  (-134.2;66.3) | 0.51 | -8.6  (-119.1;101.9) | 0.88 |
| **25-29** | 362 | 51 | 458.2  (287.1) | Ref. |  | Ref. |  |
| **30-34** | 669 | 129 | 456.8  (285.4) | -1.5  (-37.9;35.0) | 0.94 | -19.1  (-59.2;21.0) | 0.35 |
| **35-39** | 464 | 137 | 440.8  (275.2) | -17.5  (-56.7;21.7) | 0.38 | -31.7  (-79.7;16.3) | 0.20 |
| **40-45** | 241 | 76 | 465.1  (283.9) | 6.9  (-39.6;53.3) | 0.77 | -26.8  (-85.1:31.5) | 0.37 |
| **46-99** | 82 | 23 | 513.1  (337.6) | 54.8  (-13.5;123.1) | 0.12 | 8.7  (-72.7;90.1) | 0.83 |
| **Total** | 1852 | 420 |  |  |  |  |  |
| **P *overall*** |  |  |  |  | 0.38 |  | 0.77 |

Linear regression. Multiple linear regression. ^*^Men’s age at oocyte pick up, ^**^Adjusted for female age, female BMI, female smoking, diagnosis and clinic, ^1^human chorionic gonadotrophin, ^2^FET: Frozen-thawed Embryo Transfer.
