# Supplementary material for: Is paternal age associated with transfer day, developmental stage, morphology, and initial hCG-rise of the competent blastocyst leading to live birth? A multicenter cohort study
Source: PLoS One. 2022 Jul 28;17(7):e0270664. doi: 10.1371/journal.pone.0270664 (PMC9333207; doi:10.1371/journal.pone.0270664)
Supplement: S8 Table — Logistic regression. Multivariable logistic regression. Ordinal logistic regression. Ordinal multivariable logistic regression. *Men’s age at oocyte pick up, **Adjusted for male age, female BMI, female smoking, diagnosis and clinic, 1FET: Frozen-thawed Embryo Transfer, 2TE: Trophectoderm, 3ICM: Inner Cell Mass. (DOCX) [file pone.0270664.s010.docx]

**S8. Table. The association of men’s age* with stage and morphology of the competent blastocyst after FET^1^ – without 617 day 6 blastocysts**

|  | **N** | **OR** | **OR-adjusted**** |
| --- | --- | --- | --- |
| Cryopreservation day | 2798 |  |  |
| 5 | 2181 | ref. | ref. |
| 6 | 617 | **1.02 (1.00;1.03)** | 1.01 (0.98;1.03) |
| Stage (3-6) | 2766 | 1.00 (0.99;1.01) | 0.99 (0.98;1.01) |
| missing | 32 |  |  |
| TE^2^ (A-C) | 2637 | 1.02 **(1.01;1.03)** | 0.99 (0.98;1.01) |
| missing | 161 |  |  |
| ICM^3^ (A-C) | 2637 | 1.01 **(1.00;1.03)** | 0.99 (0.98;1.01) |
| missing | 161 |  |  |
| Group (1-3) | 2637 | 1.01 **(1.00;1.02)** | 0.99 (0.98;1.01) |
| missing | 161 |  |  |

*Logistic regression. Multivariable logistic regression. Ordinal logistic regression. Ordinal multivariable logistic regression. *Men’s age at oocyte pick up, **Adjusted for male age, female BMI, female smoking, diagnosis and clinic, ^1^FET: Frozen-thawed Embryo Transfer, ^2^TE: Trophectoderm, ^3^ICM: Inner Cell Mass.*
