# Supplementary material for: Is paternal age associated with transfer day, developmental stage, morphology, and initial hCG-rise of the competent blastocyst leading to live birth? A multicenter cohort study
Source: PLoS One. 2022 Jul 28;17(7):e0270664. doi: 10.1371/journal.pone.0270664 (PMC9333207; doi:10.1371/journal.pone.0270664)
Supplement: S9 Table — Linear regression. Multivariable linear regression. *Men’s age at oocyte pick up, **Adjusted for female age, female BMI, female smoking, diagnosis and clinic, 1human chorionic gonadotrophin, 2COS: Controlled Ovarian Stimulation. (DOCX) [file pone.0270664.s011.docx]

**S9 Table. The association of men’s age^*^ with implantation, initial hCG^1^ rise, of the competent blastocyst after COS^2^ - without 43 day 6 blastocysts**

| **Male age (years)** | **N** | **Missing** | **Mean hCG^2^ (sd)** | **Meandiff. (95%CI)** | **P-value** | **Adj. meandiff.**  **(95%CI)^**^** | **P-adj** |
| --- | --- | --- | --- | --- | --- | --- | --- |
| **21-24** | 27 | 0 | 346.1  (159.5) | -21.1  (-97.6;55.5) | 0.59 | -7.1  (-86.0:71.8) | 0.86 |
| **25-29** | 289 | 40 | 367.2  (197.6) | Ref. |  | Ref. |  |
| **30-34** | 623 | 107 | 329.4  (184.1) | -37.8  (-64.9;-10.8) | **0.01** | -51.6  (-80.4;-22.7) | **<0.001** |
| **35-39** | 483 | 96 | 342.9  (207.9) | -24.3  (-52.6;3.9) | 0.09 | -38.2  (-72.3;-4.2) | **0.03** |
| **40-45** | 200 | 45 | 330.4  (172.2) | -36.8  (-71.8;.-1.9) | **0.04** | -57.4  (-99.1;-15.6) | **0.01** |
| **46-99** | 77 | 14 | 367.2  (226.7) | -0.03  (-48.8;48.7) | 0.99 | -21.6  (-75.9;32.6) | 0.43 |
| **Total** | 1699 | 302 |  |  |  |  |  |
| **P *overall*** |  |  |  |  | 0.09 |  | **0.01** |

*Linear regression. Multivariable linear regression. ^*^Men’s age at oocyte pick up, ^**^Adjusted for female age, female BMI, female smoking, diagnosis and clinic, ^1^human chorionic gonadotrophin, ^2^COS: Controlled Ovarian Stimulation.*
