# Supplementary material for: Is paternal age associated with transfer day, developmental stage, morphology, and initial hCG-rise of the competent blastocyst leading to live birth? A multicenter cohort study
Source: PLoS One. 2022 Jul 28;17(7):e0270664. doi: 10.1371/journal.pone.0270664 (PMC9333207; doi:10.1371/journal.pone.0270664)
Supplement: S10 Table — Linear regression. Multivariable linear regression. *Men’s age at oocyte pick up, **Adjusted for female age, female BMI, female smoking, diagnosis and clinic, 1human chorionic gonadotrophin, 2FET: Frozen-thawed Embryo Transfer. (DOCX) [file pone.0270664.s012.docx]

**S10 Table. The association of men’s age^*^ with implantation, initial hCG^1^ rise, of the competent blastocyst after FET^2^ – without 617 day 6 blastocysts**

| **Women age^*^ (years)** | **N** | **Missing** | **Mean hCG (sd)** | **Meandiff. (95%CI)** | **P-value** | **Adj. meandiff.**  **(95%CI)^**^** | **P-adj** |
| --- | --- | --- | --- | --- | --- | --- | --- |
| **21-24** | 39 | 6 | 424.3  (246.1) | -27.1  (-122.0;67.7) | 0.58 | -12.3  (-114.1;89.4) | 0.81 |
| **25-29** | 376 | 56 | 451.4  (284.1) | Ref. |  | Ref. |  |
| **30-34** | 659 | 118 | 456.9  (281.1) | 5.5  (-30.9;42.0) | 0.77 | -7.4  (-47.3;32.5) | 0.72 |
| **35-39** | 398 | 140 | 447.5  (290.9) | -3.9  (-44.5;36.6) | 0.85 | -9.9  (-58.8;38.9) | 0.69 |
| **40-45** | 220 | 66 | 467.0  (296.5) | 15.6  (-32.2;63.5) | 0.52 | -15.1  (-73.6;43.4) | 0.61 |
| **46-99** | 83 | 20 | 508.0  (325.9) | 56.6  (-11.7;125.0) | 0.10 | -1.2  (-82.1;79.7) | 0.98 |
| **Total** | 1775 | 406 |  |  |  |  |  |
| **P *overall*** |  |  |  |  | 0.55 |  | 0.99 |

*Linear regression. Multivariable linear regression. ^*^Men’s age at oocyte pick up, ^**^Adjusted for female age, female BMI, female smoking, diagnosis and clinic, ^1^human chorionic gonadotrophin, ^2^FET: Frozen-thawed Embryo Transfer.*
