# Supplementary material for: Is paternal age associated with transfer day, developmental stage, morphology, and initial hCG-rise of the competent blastocyst leading to live birth? A multicenter cohort study
Source: PLoS One. 2022 Jul 28;17(7):e0270664. doi: 10.1371/journal.pone.0270664 (PMC9333207; doi:10.1371/journal.pone.0270664)
Supplement: S11 Table — Logistic regression. Multivariable logistic regression. Ordinal logistic regression. Ordinal multivariable logistic regression. *Men’s age at oocyte pick up, **Adjusted for female age, male BMI, male smoking, diagnosis and clinic, 1COS: Controlled Ovarian Stimulation 2TE: Trophectoderm, 3ICM: Inner Cell Mass. (DOCX) [file pone.0270664.s013.docx]

**S11 Table. The association of men’s age^*^ with stage and morphology of the competent blastocyst after COS^1^**

|  | **N** | **OR** | **OR-adjusted^**^** |
| --- | --- | --- | --- |
| **Age^*^** |  |  |  |
| **Transfer day** | 2044 |  |  |
| **5** | 2001 | ref. | ref. |
| **6** | 43 | **1.05 (1.01;1.10)** | 1.04 (0.95;1.14) |
| **Stage (3-6)** | 1979 | 0.99 (0.98;1.01) | 1.01 (0.99;1.03) |
| missing | 65 |  |  |
| **TE^2^ (A-C)** | 1959 | 1.00 (0.99;1.02) | 1.00 (0.97;1.03) |
| missing | 85 |  |  |
| **ICM^3^ (A-C)** | 1959 | 1.00 (0.99;1.02) | 0.99 (0.96;1.02) |
| missing | 85 |  |  |
| **Group (1-3)** | 1959 | 0.99 (0.98;1.01) | 1.00 (0.98;1.02) |
| missing | 85 |  |  |

*Logistic regression. Multivariable logistic regression. Ordinal logistic regression. Ordinal multivariable logistic regression. ^*^Men’s age at oocyte pick up, ^**^Adjusted for female age, male BMI, male smoking, diagnosis and clinic, ^1^COS: Controlled Ovarian Stimulation ^2^TE: Trophectoderm, ^3^ICM: Inner Cell Mass.*
