# Supplementary material for: Is paternal age associated with transfer day, developmental stage, morphology, and initial hCG-rise of the competent blastocyst leading to live birth? A multicenter cohort study
Source: PLoS One. 2022 Jul 28;17(7):e0270664. doi: 10.1371/journal.pone.0270664 (PMC9333207; doi:10.1371/journal.pone.0270664)
Supplement: S12 Table — Logistic regression. Multiple logistic regression. Ordinal logistic regression. Ordinal multiple logistic regression. *Men’s age at oocyte pick up, **Adjusted for female age, male BMI, male smoking, diagnosis and clinic, 1FET: Frozen-thawed Embryo Transfer, 2TE: Trophectoderm, 3ICM: Inner Cell Mass. (DOCX) [file pone.0270664.s014.docx]

**S12 Table. The association of men’s age^*^ with stage and morphology of the competent blastocyst after FET^1^**

|  | **N** | **OR** | **OR-adjusted^**^** |
| --- | --- | --- | --- |
| **Age^*^** |  |  |  |
| **Cryopreservation day** | 2798 |  |  |
| **5** | 2181 | ref. | ref. |
| **6** | 617 | **1.02 (1.00;1.03)** | 1.00 (0.97;1.03) |
| **Stage (3-6)** | 2766 | 1.00 (0.99;1.01) | 1.01 (0.99;1.03) |
| missing | 32 |  |  |
| **TE^2^ (A-C)** | 2637 | **1.02 (1.01;1.03)** | 1.00 (0.98;1.02) |
| missing | 161 |  |  |
| **ICM^3^ (A-C)** | 2637 | **1.01 (1.00;1.03)** | 0.99 (0.97;1.01) |
| missing | 161 |  |  |
| **Group (1-3)** | 2637 | **1.01 (1.00;1.02)** | 1.01 (0.99;1.03) |
| missing | 161 |  |  |

*Logistic regression. Multiple logistic regression. Ordinal logistic regression. Ordinal multiple logistic regression. ^*^Men’s age at oocyte pick up, ^**^Adjusted for female age, male BMI, male smoking, diagnosis and clinic, ^1^FET: Frozen-thawed Embryo Transfer, ^2^TE: Trophectoderm, ^3^ICM: Inner Cell Mass.*
