# Supplementary material for: Is paternal age associated with transfer day, developmental stage, morphology, and initial hCG-rise of the competent blastocyst leading to live birth? A multicenter cohort study
Source: PLoS One. 2022 Jul 28;17(7):e0270664. doi: 10.1371/journal.pone.0270664 (PMC9333207; doi:10.1371/journal.pone.0270664)
Supplement: S13 Table — Linear regression. Multiple linear regression. *Men’s age at oocyte pick up, **Adjusted for female age, male BMI, male smoking, diagnosis and clinic, 1human chorionic gonadotrophin, 2COS: Controlled Ovarian Stimulation. (DOCX) [file pone.0270664.s015.docx]

**S13 Table. The association of men’s age^*^ with implantation, initial hCG^1^ rise, of the competent blastocyst after COS^2^**

| **Women age^*^ (years)** | **N** | **Missing** | **Mean hCG^2^ (sd)** | **Meandiff. (95%CI)** | **P-value** | **Adj. meandiff.**  **(95%CI)^**^** | **P-adj** |
| --- | --- | --- | --- | --- | --- | --- | --- |
| **18-24** | 28 | 0 | 344.9 (156.7) | -22.3  (-97.5;52.9) | 0.56 | -19.3  (-113.0;74.5) | 0.69 |
| **25-29** | 289 | 40 | 367.2 (197.6) | ref. |  | ref. |  |
| **30-34** | 631 | 115 | 328.9 (184.1) | -38.3  (-65.3;-11.3) | **0.01** | -67.6  (-102.0;-33.0) | **<0.001** |
| **35-39** | 492 | 102 | 341.2 (206.9) | -26.0  (-54.1;2.2) | 0.07 | -59.0  (-100.4;-17.6) | **0.01** |
| **40-45** | 204 | 48 | 331.5 (172.4) | -35.7  (-70.5;-1.0) | **0.04** | -57.5  (-108.0;-6.9) | **0.03** |
| **46-99** | 80 | 15 | 370.3 (228.5) | 3.1  (-44.9;51.1) | 0.90 | -19.0  (-79.6;41.6) | 0.54 |
| **Total** | 1724 | 320 |  |  |  |  |  |
| **P *overall*** |  |  |  |  | 0.07 |  | **0.004** |

Linear regression. Multiple linear regression. ^*^Men’s age at oocyte pick up, ^**^Adjusted for female age, male BMI, male smoking, diagnosis and clinic, ^1^human chorionic gonadotrophin, ^2^COS: Controlled Ovarian Stimulation.
