# Supplementary material for: Is paternal age associated with transfer day, developmental stage, morphology, and initial hCG-rise of the competent blastocyst leading to live birth? A multicenter cohort study
Source: PLoS One. 2022 Jul 28;17(7):e0270664. doi: 10.1371/journal.pone.0270664 (PMC9333207; doi:10.1371/journal.pone.0270664)
Supplement: S14 Table — Linear regression. Multiple linear regression. *Men’s age at oocyte pick up, **Adjusted for female age, male BMI, male smoking, diagnosis and clinic, 1human chorionic gonadotrophin, 2FET: Frozen-thawed Embryo Transfer. (DOCX) [file pone.0270664.s016.docx]

**S14 Table. The association of men’s age^*^ with implantation, initial hCG^1^ rise, of the competent blastocyst after FET^2^**

| **Women age^*^ (years)** | **N** | **Missing** | **Mean hCG (sd)** | **Meandiff. (95%CI)** | **P-value** | **Adj. meandiff.**  **(95%CI)^**^** | **P-adj** |
| --- | --- | --- | --- | --- | --- | --- | --- |
| **18-24** | 46 | 6 | 420.7 (240.7) | -28.4  (-115.0;58.2) | 0.52 | -98.3  (-211.0;14.3) | 0.09 |
| **25-29** | 461 | 63 | 449.2 (277.1) | ref. |  | ref. |  |
| **30-34** | 839 | 148 | 465.2 (289.9) | 16.1  (-16.4;48.6) | 0.33 | 13.0  (-29.4;55.3) | 0.55 |
| **35-39** | 555 | 166 | 441.2 (278.7) | -8.0  (-43.2;27.3) | 0.66 | -11.5  (-62.7;39.6) | 0.66 |
| **40-45** | 294 | 89 | 465.7 (287.9) | 16.6  (-25.2;58.4) | 0.44 | -10.1  (-72.0;51.9) | 0.75 |
| **46-99** | 105 | 26 | 510.8 (331.5) | 61.7  (1.1;122.2) | **0.04** | 7.7  (-78.5;94.0) | 0.86 |
| **Total** | 2300 | 498 |  |  |  |  |  |
| **P *overall*** |  |  |  |  | 0.19 |  | 0.40 |

Linear regression. Multiple linear regression. ^*^Men’s age at oocyte pick up, ^**^Adjusted for female age, male BMI, male smoking, diagnosis and clinic, ^1^human chorionic gonadotrophin, ^2^FET: Frozen-thawed Embryo Transfer.
