# Supplementary material for: Is paternal age associated with transfer day, developmental stage, morphology, and initial hCG-rise of the competent blastocyst leading to live birth? A multicenter cohort study
Source: PLoS One. 2022 Jul 28;17(7):e0270664. doi: 10.1371/journal.pone.0270664 (PMC9333207; doi:10.1371/journal.pone.0270664)
Supplement: S15 Table — (DOCX) [file pone.0270664.s017.docx]

**S15 Table. Analysis of interaction between women age (age) and male age (age_vir)**

|  | COS+FET age_vir*age  p-value |
| --- | --- |
| Transfer day  *(multivariable logistic regression)* | 0.34 |
| Stage  *(multivariable ordinal logistic regression)* | 0.29 |
| Trophectoderm  *(multivariable ordinal logistic regression)* | 0.46 |
| Inner Cell Mass  *(multivariable ordinal logistic regression)* | 0.09 |
| hCG  *(multivariable linear regression)* | 0.22 |
