# Supplementary material for: Is paternal age associated with transfer day, developmental stage, morphology, and initial hCG-rise of the competent blastocyst leading to live birth? A multicenter cohort study
Source: PLoS One. 2022 Jul 28;17(7):e0270664. doi: 10.1371/journal.pone.0270664 (PMC9333207; doi:10.1371/journal.pone.0270664)
Supplement: Attachment — Submitted filename: Response to Reviewers.docx [file pone.0270664.s018.docx]

Review

PONE-D-21-32713

Is paternal age associated with transfer day, developmental stage, morphology, and initial hCG-rise of the competent blastocyst leading to live birth? a multicenter cohort study

PLOS ONE

# Journal Requirements:

When submitting your revision, we need you to address these additional requirements.

1. Please ensure that your manuscript meets PLOS ONE's style requirements, including those for file naming. The PLOS ONE style templates can be found at 
<https://journals.plos.org/plosone/s/file?id=wjVg/PLOSOne_formatting_sample_main_body.pdf> and 
<https://journals.plos.org/plosone/s/file?id=ba62/PLOSOne_formatting_sample_title_authors_affiliations.pdf>

*Reply: Thank you for the reminder. We have now ensured that our manuscript meets the requirements.*

2. We note that you have included the phrase “data not shown” in your manuscript. Unfortunately, this does not meet our data sharing requirements. PLOS does not permit references to inaccessible data. We require that authors provide all relevant data within the paper, Supporting Information files, or in an acceptable, public repository. Please add a citation to support this phrase or upload the data that corresponds with these findings to a stable repository (such as Figshare or Dryad) and provide and URLs, DOIs, or accession numbers that may be used to access these data. Or, if the data are not a core part of the research being presented in your study, we ask that you remove the phrase that refers to these data.

*Reply: Instead of inclusion of the phrase “data not shown” we have referred to and uploaded the results as supporting tables. Please see the uploads, the references in the section of the Results and the section of Supporting information (in the main document after the references).*

3. Thank you for submitting the above manuscript to PLOS ONE. During our internal evaluation of the manuscript, we found significant text overlap between your submission and the following previously published works, some of which you are an author.

<https://bmcpregnancychildbirth.biomedcentral.com/articles/10.1186/s12884-020-03348-2>
<https://www.fertstert.org/article/S0015-0282(20)32191-9/fulltext>

We would like to make you aware that copying extracts from previous publications, especially outside the methods section, word-for-word is unacceptable. In addition, the reproduction of text from published reports has implications for the copyright that may apply to the publications.

Please revise the manuscript to rephrase the duplicated text, cite your sources, and provide details as to how the current manuscript advances on previous work. Please note that further consideration is dependent on the submission of a manuscript that addresses these concerns about the overlap in text with published work.

We will carefully review your manuscript upon resubmission, so please ensure that your revision is thorough

*Reply: Thank you for pointing out that you have found some text overlap in two articles.*

*The second article that you link to was written by our research group. The article has female age as exposure, and this manuscript that we uploaded to PLOSONE has male age as exposure. The article on female age was made parallel to the male age article. The outcomes in the two articles are the same. By examining the comparability between the two articles electronically, we can see that in the section of the Methods there are some small overlaps, which much be expected, since the same data and the same methodology was applied. For example in the sections “Treatment regimen” / ”Clinical setting”, “Outcomes” , “Covariates” and less in the section “Statical methods”. However, beyond these methodological issues, there is no overlap in the sections: “Introduction, “Results” and “Discussion”. Furthermore, we have referred to the article of female age in line, 102-104, 121, 196, 366, 368, 370, 383, 385, 386 and 390 in the clean version.*

*The first article that you link to has a very different topic of interest (Prevalence and associated factors of birth asphyxia among live births at Debre Tabor General Hospital, North Central Ethiopia). As we do not include data from the children it is difficult to see where the overlap should be, also when we read the paper.*

Please review your reference list to ensure that it is complete and correct. If you have cited papers that have been retracted, please include the rationale for doing so in the manuscript text, or remove these references and replace them with relevant current references. Any changes to the reference list should be mentioned in the rebuttal letter that accompanies your revised manuscript. If you need to cite a retracted article, indicate the article’s retracted status in the References list and also include a citation and full reference for the retraction notice.

*Reply: We have reviewed all the references and checked that none of them have been retracted.*

Additional Editor Comments (if provided):

The paper has been appreciated by the two Reviewers. However, there are some minor aspects that need to be addressed:
1. The Authors has underlined the importance of the results of the fresh transfer on day 6 compared to day 5. However, the number of transfers in day 6 was only 43. How can the authors be so sure not to have a type 1 error? The Authors may discuss this aspect. The manuscript should be also modified according to this possible bias.

*Reply: Unfortunately, it is by definition not possible to be sure that a type 1 error is avoided, as there will always be a risk of that type of error. The low number of day 6 transfers is reflected in the confidence interval of the adjusted OR: 1.06, CI (1.00;1.13) where the precision of the estimate is showed. We have added a sentence in the Discussion. Please see line 328-329 in the clean version.*

2. The strategy to transfer a fresh embryo in day 6 is not so diffuse given the idea that in day 6 the endometrium is less receptive. As a matter of fact, the number of fresh transfers in day 6 is low. The Authors stated that they have corrected for centers but results are not shown. Is it possible that few centers adopted this strategy and they had older patients?

*Reply: Thank you for that comment. We have adjusted for center by inclusion of “Clinic” in all our multivariable analyses. Please see the legends in Table 2 and Table 3.*

3. Was there some kind of standardization of blastocyst morphology among centers?

*Reply: Thank you for the comment.* *To reduce the risk of interobserver and intraobserver variation, most of the participating clinics have a standardized training set-up planned annually, which is mandatory to participate in. However, some variation may exist between the IVF laboratories, and therefore the multivariable analyses were adjusted for clinic. We added a sentence about this in the section of Materials and Methods. Please see line 166-168 in the clean version.*

4. Given the small ORs, the conclusions seem overemphasized.

*Reply: We highlight the adjusted association between male age and transfer day OR* *1.06, CI (1.00;1.13), which means that with a one year increase in male age there is a 6% increased probability that the competent blastocyst will be transferred on day 6 compared to day 5. An assessment of whether this estimate is overemphasized is very relevant. The relevance per one year increase in male age is relatively small, but one should bear in mind that looking at the estimate over several years it may sum up to fairly high risk. Further, our result should be interpreted with caution, as OR overestimates the true risk ratio, particularly in case of frequent outcomes. We have added a sentence with that modification in the Discussion. Please see line 317-319 and line 326-328 in the clean version. Further we have modified the conclusions. Please see line 63-64 and line 391-393 in the clean version.*

5. The choice of the bHCG levels is debatable. Please add some references to support this choice.

*Reply: We do not entirely understand this comment. We have not chosen any beta-hCG-levels, and we do not report any cut-off values for hCG or anything similar. We merely compare the observed (standardized) hCG-levels.*

Reviewers' comments:

Reviewer's Responses to Questions

**Comments to the Author**

1. Is the manuscript technically sound, and do the data support the conclusions?

The manuscript must describe a technically sound piece of scientific research with data that supports the conclusions. Experiments must have been conducted rigorously, with appropriate controls, replication, and sample sizes. The conclusions must be drawn appropriately based on the data presented.

Reviewer #1: Yes

Reviewer #2: Yes

2. Has the statistical analysis been performed appropriately and rigorously?

Reviewer #1: Yes

Reviewer #2: Yes

3. Have the authors made all data underlying the findings in their manuscript fully available?

The [PLOS Data policy](http://www.plosone.org/static/policies.action#sharing) requires authors to make all data underlying the findings described in their manuscript fully available without restriction, with rare exception (please refer to the Data Availability Statement in the manuscript PDF file). The data should be provided as part of the manuscript or its supporting information, or deposited to a public repository. For example, in addition to summary statistics, the data points behind means, medians and variance measures should be available. If there are restrictions on publicly sharing data—e.g. participant privacy or use of data from a third party—those must be specified.

Reviewer #1: Yes

Reviewer #2: Yes

4. Is the manuscript presented in an intelligible fashion and written in standard English?

PLOS ONE does not copyedit accepted manuscripts, so the language in submitted articles must be clear, correct, and unambiguous. Any typographical or grammatical errors should be corrected at revision, so please note any specific errors here.

Reviewer #1: Yes

Reviewer #2: Yes

5. Review Comments to the Author

Please use the space provided to explain your answers to the questions above. You may also include additional comments for the author, including concerns about dual publication, research ethics, or publication ethics. (Please upload your review as an attachment if it exceeds 20,000 characters)

Reviewer #1: I must congratulate you for the very interesting manuscript, which addresses the effect of paternal age on embryonic development and the chance of giving rise to a pregnancy.
The data shown will help to better understand the effect of paternal age on ART results.

Reviewer #2: Very nice paper and good numbers. It’s important to publish this kind of paper in order to have good data to explain our patients about impact of paternal age in reproductive medicine. Congratulations and hopefully will help many others physicians.

6. PLOS authors have the option to publish the peer review history of their article ([what does this mean?](https://journals.plos.org/plosone/s/editorial-and-peer-review-process#loc-peer-review-history)). If published, this will include your full peer review and any attached files.


If you choose “no”, your identity will remain anonymous but your review may still be made public.


**Do you want your identity to be public for this peer review?** For information about this choice, including consent withdrawal, please see our [Privacy Policy](https://www.plos.org/privacy-policy).

Reviewer #1: **Yes:** Javier Garcia-Ferreyra PhD

Reviewer #2: **Yes:** Alessandro Schuffner

[NOTE: If reviewer comments were submitted as an attachment file, they will be attached to this email and accessible via the submission site. Please log into your account, locate the manuscript record, and check for the action link "View Attachments". If this link does not appear, there are no attachment files.]

While revising your submission, please upload your figure files to the Preflight Analysis and Conversion Engine (PACE) digital diagnostic tool, <https://pacev2.apexcovantage.com/>. PACE helps ensure that figures meet PLOS requirements. To use PACE, you must first register as a user. Registration is free. Then, login and navigate to the UPLOAD tab, where you will find detailed instructions on how to use the tool. If you encounter any issues or have any questions when using PACE, please email PLOS at [figures@plos.org](mailto:figures@plos.org). Please note that Supporting Information files do not need this step.

*In compliance with data protection regulations, you may request that we remove your personal registration details at any time.* [*(Remove my information/details)*](https://www.editorialmanager.com/pone/login.asp?a=r)
